# Dynamic Changes in Subgraph Preference Profiles of Crucial Transcription Factors

**DOI:** 10.1371/journal.pcbi.0020047

**Published:** 2006-05-12

**Authors:** Zhihua Zhang, Changning Liu, Geir Skogerbø, Xiaopeng Zhu, Hongchao Lu, Lan Chen, Baochen Shi, Yong Zhang, Jie Wang, Tao Wu, Runsheng Chen

**Affiliations:** 1 Bioinformatics Laboratory and National Laboratory of Biomacromolecules, Institute of Biophysics, Chinese Academy of Sciences, Beijing, China; 2 Bioinformatics Research Group, Key Laboratory of Intelligent Information Processing, Institute of Computing Technology, Chinese Academy of Sciences, Beijing, China; 3 Graduate School of the Chinese Academy of Sciences, Beijing, China; MRC Laboratory of Molecular Biology, United Kingdom

## Abstract

Transcription factors with a large number of target genes—transcription hub(s), or THub(s)—are usually crucial components of the regulatory system of a cell, and the different patterns through which they transfer the transcriptional signal to downstream cascades are of great interest. By profiling normalized abundances (A_N_) of basic regulatory patterns of individual THubs in the yeast Saccharomyces cerevisiae transcriptional regulation network under five different cellular states and environmental conditions, we have investigated their preferences for different basic regulatory patterns. Subgraph-normalized abundances downstream of individual THubs often differ significantly from that of the network as a whole, and conversely, certain over-represented subgraphs are not preferred by any THub. The THub preferences changed substantially when the cellular or environmental conditions changed. This switching of regulatory pattern preferences suggests that a change in conditions does not only elicit a change in response by the regulatory network, but also a change in the mechanisms by which the response is mediated. The THub subgraph preference profile thus provides a novel tool for description of the structure and organization between the large-scale exponents and local regulatory patterns.

## Synopsis

Transcription factors are proteins that bind to short segments of DNA, thereby controlling transcription and expression of other genes. Transcription factors may control a number of other genes, and in turn be controlled by other transcription factors, thus forming an extensive transcriptional network of control and counter-control, which acts through space and time in the cell. In transcriptional networks, transcription factors and their target genes form various patterns (called subgraphs or motifs) that are suspected of being of importance to how transcription factors exert their control of cellular processes. Zhang and colleagues have studied how a subset of transcription factors (called transcription hubs) utilizes such subgraphs in networks generated from yeast cells under various cellular states and environmental conditions. Their analyses show that different transcription hubs in the same network prefer different types of subgraphs, and that these preferences are not governed by subgraph frequencies in the network. They further show that when cellular conditions change, the transcription hubs frequently change their subgraph preferences, indicating that different modes of control require different types of subgraph use. These findings could have implications for our understanding of the mechanisms that underlie the fine-tuned control systems that govern a cell or an organism.

## Introduction

The study of transcriptional regulatory networks is of central importance to post-genomic research, because every cell is the product of specific programs involving regulated transcription of a large number of genes. With increasing amounts of data becoming available by advanced data collection and analysis methods, network models have been established in a number of different species [1,2]. Transcriptional regulatory networks can be depicted as directed graphs, in which transcription factors and their target genes are represented as vertices, whereas the binding of a transcription factor in the regulatory region of a gene is represented as a directed edge. The transcriptional relationship between several transcription factors and their regulated genes is represented as multi-node subgraphs of the network graph. Some of the subgraph patterns are immediately biologically meaningful, including the feed-forward loop (FFL), feedback loop (FBL), single input motif (SIM), and multi-input motif [1–3]. Such patterns usually exert specific regulatory capacities, for example, a SIM may be used for coordinating a set of genes, whereas a FFL has the potential to provide temporal control of a process [1,2]. However, subgraphs do not represent independent units that are functionally separable from the rest of the network. Subgraphs are likely to aggregate with other subgraphs around some highly connected transcription factors [4]; an individual transcription factor can thus be a member of many different subgraph patterns with different connectivity. At the global level, analysis of the network topological organization shows that most target genes are regulated by a small number of factors. On the other hand, the number of target genes regulated by a given transcription factor is distributed according to power law, indicating that a selected few transcription factors participate in the regulation of a disproportionately large number of target genes [5]. This particular type of well-connected transcription factor has been called a “transcription hub (THub),” and it is usually representative of crucial and essential transcription factors in an organism [6].

Transcriptional regulatory networks have evolved to process information such as external nutrients and stress [7], and the way that the transcription factors in a network perform will necessarily differ extensively. Analysis of signal transduction in a mammalian cellular network showed that three ligands—glutamate, norepinephrine, and brain-derived neurotrophic factor—make use of different types of sub-patterns at different levels of the network, and at different subgraph densities [8]. Similarly, different condition-specific sub-networks of the yeast transcriptional regulation network showed different frequencies of various regulatory patterns, and substantial changes in network structure occurred in response to changes in environment and during the development of the organism [9]. However, so far, very few studies have documented how differences in regulatory motif abundance relate to individual transcription factors on a genome-wide basis.

**Figure 1:**
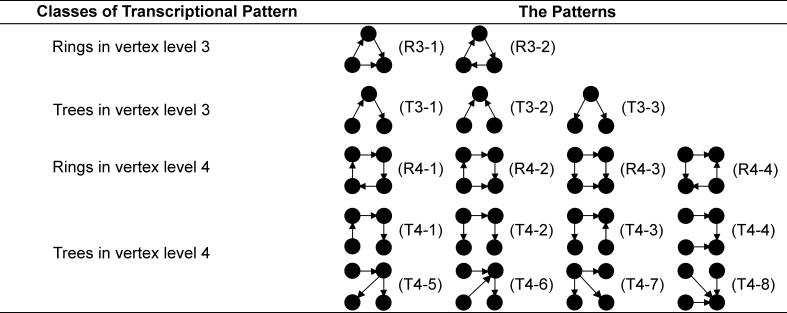
The Basic Subgraphs at the 3- and 4-Vertex Level The IDs of each subgraph are given in brackets.

We have revisited the datasets of Luscombe et al. [9], in which several transcriptional sub-networks corresponding to particular cellular or environmental conditions (e.g., cell cycle, stress response, etc.) have been identified, and used these data to quantitatively depict the subgraph context in transcription factor downstream cascades. To this end, we defined the “subgraph preference profile (SPP)” of a transcription factor as the vector of the normalized abundances of a set of basic regulatory subgraph patterns, obtained by counting the weighted census of the regulatory subgraph patterns occurring downstream of the transcription factor. This set of basic regulatory subgraph patterns contains five 3-vertex patterns and 12 4-vertex patterns (Figure 1). Based on this definition we studied the relationship between network topology and transcription factor preference profiles, and the dynamic changes in preference profiles occurring among different cellular states or environmental conditions.

An algorithm was developed to analyze systematically the subgraph preferences of transcription factors in a regulatory network. Because the THubs represent the most influential and essential components in a network [6,10], we limited our interest to these crucial transcription factors. Using this algorithm, we obtained two major results: 1) Certain kinds of regulatory subgraph patterns are preferred by certain THubs in different (sub-)networks, and these preferences cannot be explained by general variations in motif abundance; 2) THubs showed dynamic changes in subgraph preferences when cellular or external conditions varied.

## Results

We have investigated the relationship between the THubs and the normalized abundances (see Materials and Methods) of a subset of regulatory subgraph patterns (Figure 1) in yeast transcriptional regulation networks. We selected the static network, and the cell cycle, sporulation, diauxic shift, DNA damage, and stress response sub-networks from Luscombe et al. [9] for analysis. A transcription factor in the static network was regarded as a THub and included in the analysis if it had 39 or more out-degrees; similarly, THubs in the condition-specific sub-networks had five or more out-degrees. This produced 50 THubs in the static network, and 29–48 THubs in the sub-networks (Table 1). Two categories of basic regulatory subgraph patterns were included, the ring and the tree, both with either three or four vertices, thus giving four sets of subgraph patterns (Figure 1).

**Table 1:**
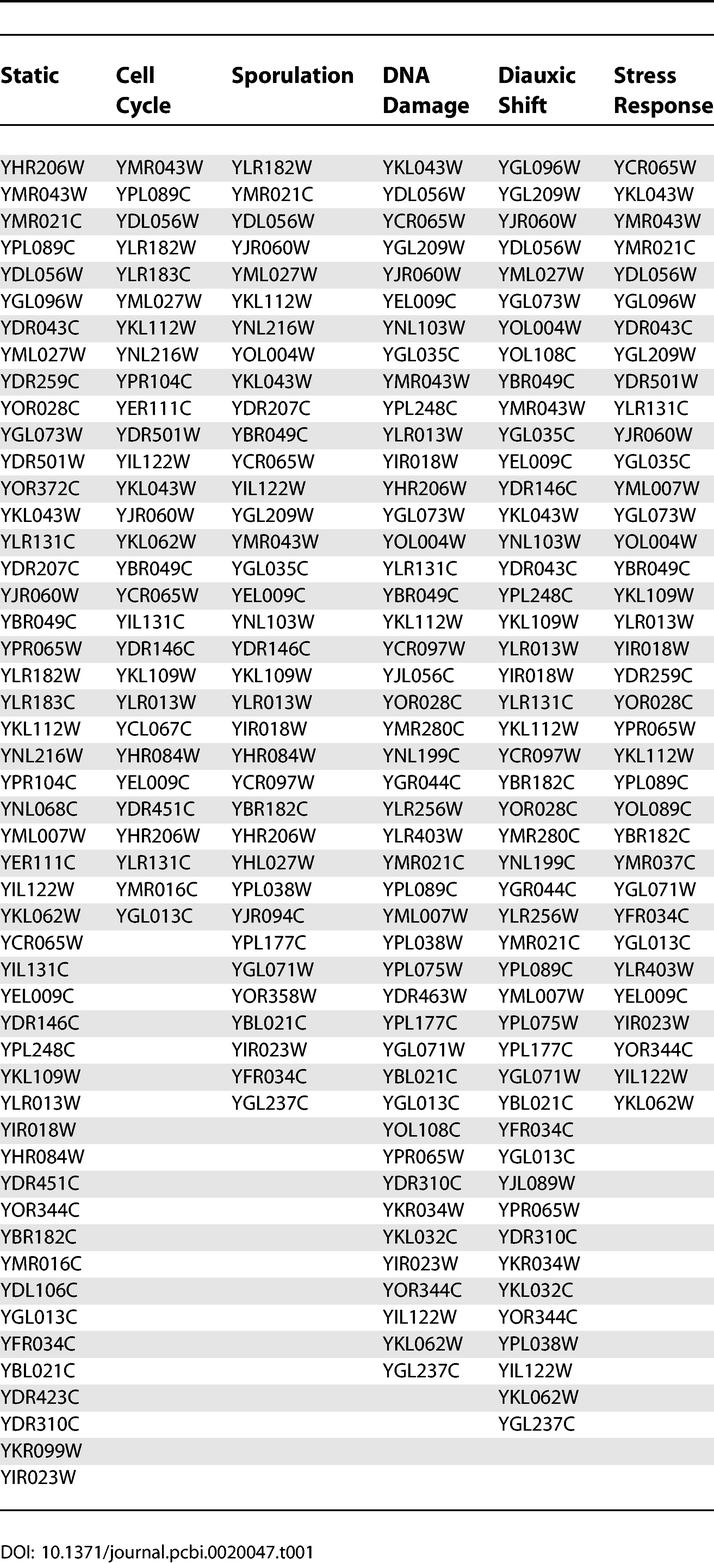
The Transcriptional Hubs Included in This Study

**Figure 2:**
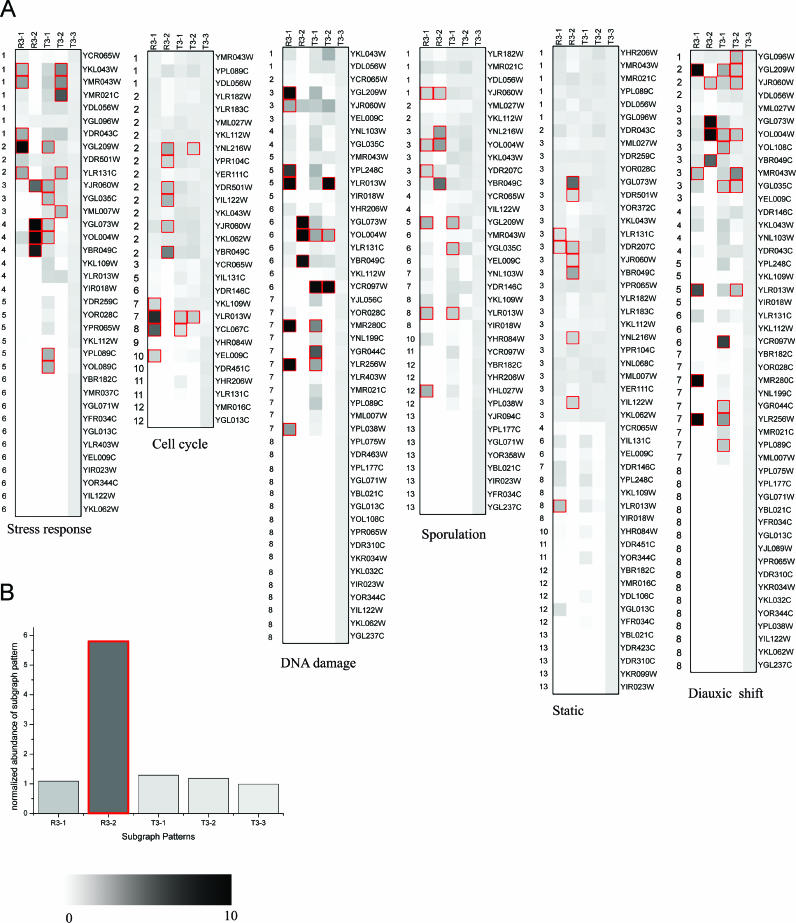
SPP and SPL (A) THub SPLs at the 3-vertex level of the yeast static transcriptional regulatory network and the five condition-specific sub-networks. The THub SPPs (rows) are ordered according to the hierarchical structure of each network. The layer numbers are given to the left of each row. The normalized abundances (A_N_) of the regulatory subgraph patterns are represented as shades of grey (black means *A_N_ ≥ 10*); squares with red borders indicating the significantly preferred patterns. The corresponding figure for the 4-vertex level SPLs can be found in Protocol S1. (B) Three-vertex SPP of the THub YGL073W.

The normalized abundance of a subgraph pattern was calculated as a weighted census of all occurrences of this pattern in the downstream cascade of a given THub, and the value of this normalized abundance was used to represent the preference of this THub for the subgraph pattern (i.e. subgraph preference). A regulatory subgraph occurring at a THub with a significantly higher normalized abundance than in the rest of the network, was termed a “preferred” regulatory subgraph pattern of this THub. The normalized abundances of the entire subset of regulatory patterns constituted the THub SPP. When, in turn, the preference profiles of all THubs in a network were assembled in a matrix, this formed a “subgraph preference landscape (SPL)” of that particular network (Figure 2).

SPLs were calculated for all six networks and sub-networks. Similar to what previously has been found in Escherichia coli [11,12], the analyses of the networks yielded a multi-layered hierarchical cascade structure (see Protocol S1). There were 14 layers in the static network, and 13, 14, 9, 9, and 7 layers, in the cell cycle, sporulation, diauxic shift, DNA damage, and stress response condition-specific sub-networks, respectively. As the SPLs were laid out according to the hierarchical cascades, the common trend for all networks was that the SPPs of THubs in the upper layers were more complex than those of THubs in the lower layers (Figure 2A). In the following sections, we first describe the preference profiles of THubs in the different networks, and we thereafter go on to describe the dynamic characteristics of the THub subgraph profiles between the five condition-specific sub-networks.

### THub Subgraph Preferences Differ among Regulatory Networks

Different THubs tended to prefer certain regulatory subgraph patterns, shown as dark squares in Figure 2A. The SIMs (T3–3 and T4–7; Figure 1) were the only regulatory subgraph patterns that appeared in all THub preference profiles and in all layers. However, these motifs were not the preferred patterns of any THub. Conversely, FFLs (R3–1) were preferred by certain number of THubs, occurring at all layers of the networks. THubs preferring FBLs (R3–2, R4–1) were, on the other hand, located in the upper levels of the cell cycle and sporulation sub-networks, but at the lower levels of three other sub-networks (Figure 2A).

Over-representation of certain regulatory subgraph patterns in the various networks could not explain the strong subgraph preferences of the THubs. The regulatory subgraph patterns FFL and SIM, also called network motifs [1–3], were all significantly over-represented in most of the transcriptional regulatory networks. However, SIMs were never preferred by any THub under any condition, and contrarily, certain sparsely represented subgraph patterns (like T3–1, T3–2) were significantly preferred by some THubs.

**Figure 3:**
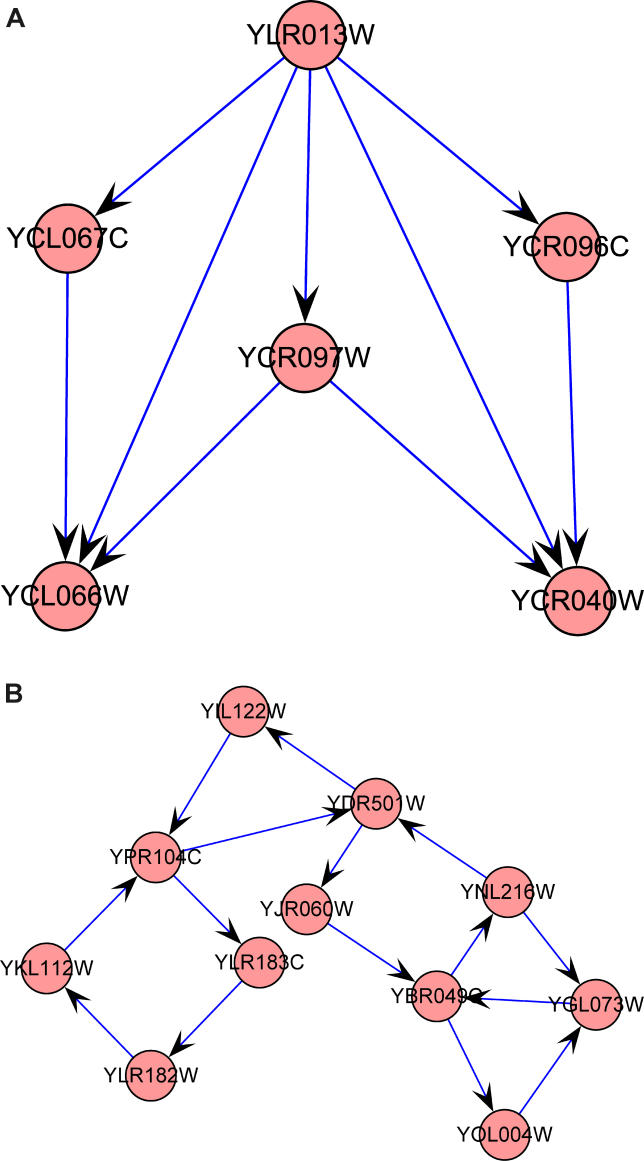
Two Examples of Yeast THubs with Significantly Preferred Regulatory Subgraph Patterns (A) The THub *YLR013W (GAT3)* significantly preferred FFLs in the cell cycle sub-network. (B) The THub *YBR049C (Reb1)* preferred FBLs in the static network.

The high preference of a regulatory subgraph pattern by a given THub might be an effect of pattern clustering. For example, the FFL was the preferred regulatory subgraph pattern by the THub *YLR013W (GAT3)* in the cell cycle specific sub-network. There are four FFLs clustered around *YLR013W,* forming a symmetrical grid (Figure 3). However, not all high regulatory SPPs can be explained by local clustering. In the entire static network, there are only three instances of 3-vertex FBLs and two instances of 4-vertex FBLs. These FBLs are not only clustered together, but also serially interlinked into a larger loop, whose vertices are all THubs (Figure 3). Despite this, not all THubs in this serial loop preferred FBLs (e.g., *YKL112W, YLR182W,* and *YLR183C; ABF1, Swi6,* and *TOS4,* respectively), and the only transcription factor in this larger loop that preferred FBLs under all conditions was *YBR049C (Reb1)*. The regulatory subgraph pattern preferences also could not be fully explained by the pattern density. The vertices of FBLs located in the upper parts of the network cascades, were directly regulated by five global regulators (*YDL056W, YMR021C, YPL038W, YMR043W,* and *YPL089C; MBP1, Mac1, Met31, Mcm1,* and *RLM1,* respectively), and 3,040 out of 3,459 genes in the static network were further regulated through these three FBLs. Transcription factors forming FBLs are often involved in fundamental biological functions, such as *YBR049C (Reb1),* which is required for termination of RNA polymerase I transcription [13]. As the pattern density surrounding a transcription factor increases nearly exponentially with an increasing number of target genes, most of FBL-preferring transcription factors have high pattern densities (e.g., *YBR049C* regulates 163 target genes); however, there are notable exceptions to this, for instance the FBL-preferring transcription factor *(YGL073W;HFS1)* in the static network regulates only 63 target genes. Thus the tendency for THubs to prefer different regulatory subgraph patterns under different conditions can neither be fully explained by the clustering of certain regulatory subgraph patterns nor by bias in the abundance of subgraph patterns, and it apparently represents an inherent characteristic of THubs toward utilizing particular types of subgraphs under specific cellular and environmental conditions.

Certain regulatory subgraph patterns, other than the globally identified motifs, were also preferred by some THubs. An example is *YMR043W (Mcm1),* a cell-type-specific transcription and pheromone response-related global transcription factor which may play a central role in the formation of both repressor and activator complexes [14]. In the stress response sub-network, this transcription factor preferred several regulatory subgraph patterns, such as the non-motif regulator chain (T4–1) and multi-pooling regulation (T4–8), in addition to the FFL motif (Table 2).

**Table 2:**
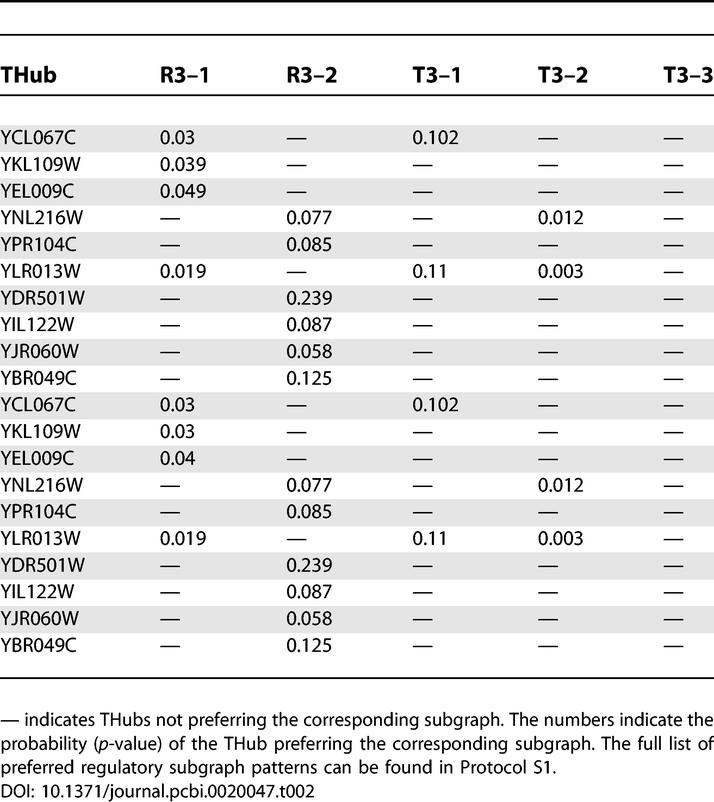
The THubs with Preferred 3-Vertices Subgraphs in the Cell Cycle Sub-Network

An attempt to relate subgraph preferences to Biological Process Annotations in the Gene Ontology [15] did not produce any firm correlations. There might be a tendency for FFL-preferring THubs and their target genes to be associated with processes related to energy generation and general metabolism; likewise, FBL-preferring THubs and target genes could possibly be associated with processes involving larger cellular structures (see Protocol S1 for details).

### Evidence of Dynamic Shifts in THub SPPs among Condition-Specific Sub-Networks

When comparing the THub preference landscapes for the five condition-specific sub-networks, we observed dynamic changes in SPPs between different conditions.

First, the SPLs changed dynamically between the different conditions. The 3-vertex preference landscapes of the diauxic shift, DNA damage, and stress response sub-networks (termed “exogenous sub-networks” by Luscombe et al. [9]) were, on one hand, distinctly different from the two “endogenous” networks (cell cycle and sporulation), except for some similarity between the sporulation and stress response sub-networks (Kolmogorov-Smirnov test; Table 3). This exception may reflect a partial exogenous influence (i.e. nitrogen depletion) during sporulation. The three 3-vertex preference landscapes of the exogenous networks were relatively similar among themselves, whereas those of the two endogenous conditions showed significant differences in distributions of normalized subgraph abundances. The differences among the 4-vertex preference landscapes were roughly in accordance with the observations made for the 3-vertex level (see Protocol S1).

**Table 3:**
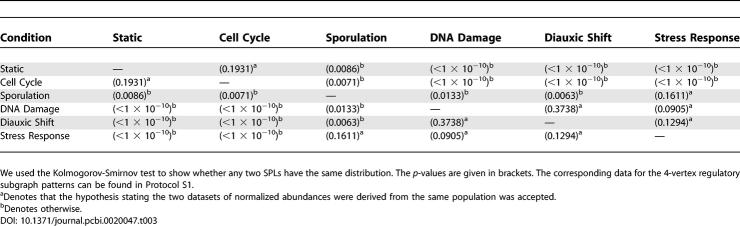
Distribution of Normalized Abundances of 3-Vertex Regulatory Subgraph Patterns under Different Conditions

Second, calculating Euclidean distances between pairs of SPPs, we show that the preference profiles of THubs within a layer had different tendencies towards similarity in the different networks. For 3-vertex subgraphs, THubs within the same layer of the static, cell cycle, or stress response networks, tended to have similar preference profiles (Table 4). On the other hand, same-layer THubs of the sporulation, DNA damage, or diauxic shift networks were more diverse in 3-vertex SPPs. At the 4-vertex level, THubs at the same-layer of the static, cell cycle, and diauxic shift networks had similar SPPs, but the preference profiles of same-layer THubs of the other networks were quite different (Protocol S1). Because the preference profiles of THubs in the bottom layer only have SIMs in any transcription regulatory network, we excluded the THubs at the bottom layer in this assessment of within-layer THub preference profile similarities.

**Table 4:**
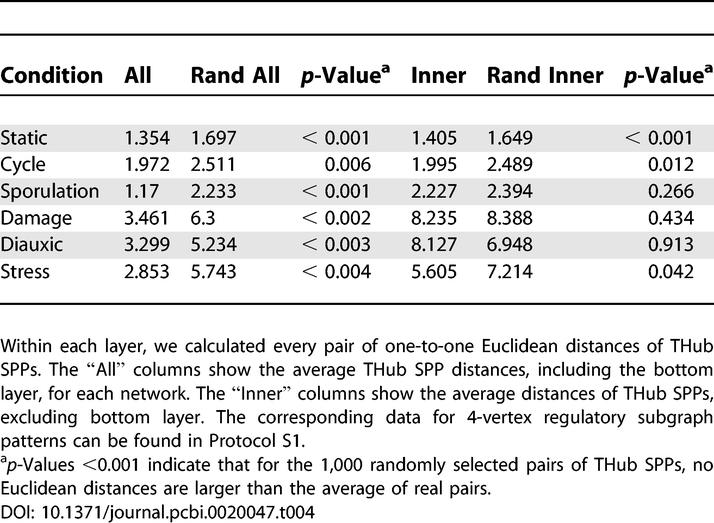
Euclidean Distances of THub SPPs (3-Vertex Level) within Each Layer

**Figure 4:**
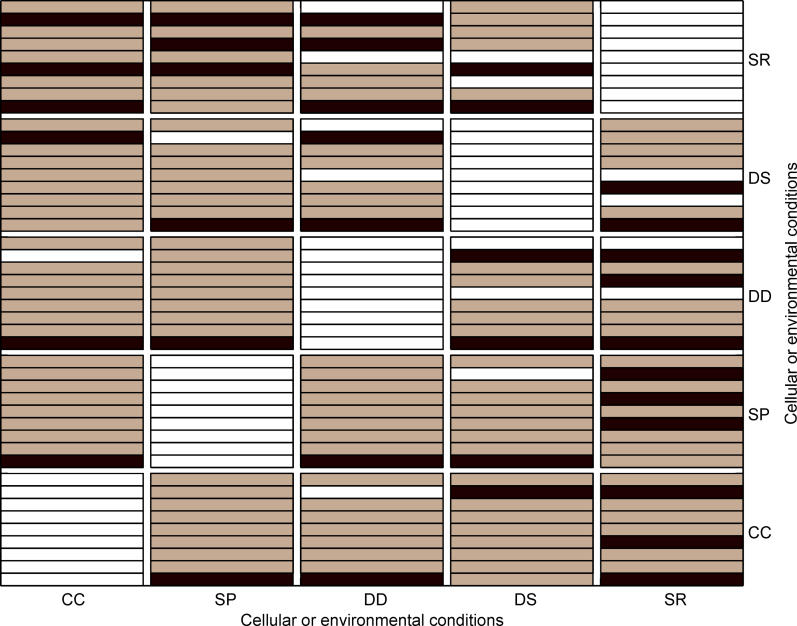
Dynamic Shifts in the SPPs (3-Vertex Level) of Nine THubs The bars represent the THubs *YDL056W, YMR043W, YBR049C, YJR060W, YIL122W, YKL043W, YKL112W, YEL009C,* and *YLR013W* (top to bottom). A black bar indicates that the THub showed a significant change in its SPP between the two conditions, whereas a white bar indicates a significant lack of change in the SPP of the THub. The assessment of the statistical significances was made by comparing to a set of random networks as described in Protocol S1.

Finally, we looked into the dynamics of the preference profiles of nine transcription factors that were identified as THubs in all five of the condition-specific sub-networks (Figure 4). Of these there was only one, *YLR013W (GAT3),* whose preference profile changed significantly between the two endogenous sub-networks*,* despite the fact that the distributions of normalized abundances of regulatory subgraph patterns in the two sub-networks were quite different. On the other hand, four of the THubs (*YMR043W, YJR060W, YKL043W,* and *YLR013W; Mcm1, CBF1, PHD1,* and *GAT3,* respectively) showed significantly altered preference profiles in the three exogenous sub-networks, even though the SPLs of these networks had similar distributions of normalized pattern abundances. At the 4-vertex level, we observed even more dynamic changes in preference profiles (see Protocol S1). These dynamic changes might reflect switches in THub biological function with altered environmental conditions. For example, in the cell cycle sub-network, a transcription factor required for nucleosome function *(YJR060W; CBF1)* [16] favored FBLs at both the 3- and 4-vertex levels. In the stress response condition, *YJR060W* also prefers FBLs, but in another adverse condition, DNA damage, *YJR060W* switches to favor FFLs. As a clock- and oscillator-like gene circuit [17], FBLs may control the cell growth rate, and the high preference for FBLsof *YJR060W* suggests that this gene may have certain clock- and oscillator-related role in the cell cycle process. The SPPs of *YJR060W* thus suggests that this transcription factor employs different mechanisms, FFLs [18], and FBLs [19] in response to changing conditions.

## Discussion

Recent cellular network studies have focused either on global topological organization, or on local structure occurrences (for review, see [10]). However, investigators need a novel tool to describe the topological and dynamic characteristics of a cellular network that appear between the global and the local level [20]. In this study, we have tried to connect the two opposite poles of cellular network research by investigating the way crucial transcription factors propagate their transcriptional signals to the downstream cascades. The subgraph patterns of regulatory transcription factors have been reported to form clusters [4], but how these clusters affect the propagation of transcriptional signals has been poorly understood. We have developed an approach to count weighted censuses of connected subgraph patterns below these well-connected THubs, which represent the most influential and essential components in a network [6,10].

To our knowledge, our work is the first to quantify the relationship between THubs and their associated regulatory subgraph patterns. The tendencies we present here have been carefully normalized against biases of the networks, and have been well-controlled against random background. The results were also strongly robust against several sources of noise, from both the ChIP-chip experimental data and the condition-specific sub-network specification.

No convincing overall associations between subgraph preferences and gene function of biological process could be found. This could be due to a number of reasons. One is that the relatively few subgraphs at the 3- and 4-vertex levels will necessarily need to be used for a variety of functions or processes going across the established patterns of gene or process annotation found in established databases. Another might be that annotations of biological function or process are (statically) linked to genes (THubs or targets), whereas the associations between THubs, subgraphs, and target genes are, as our data indicate, dynamic, varying between different cellular conditions, thus rendering any possible association between subgraph (preference) and biological process very difficult to capture by analysis of present-day databases. In any case, the analysis of subgraph—transcription factor—target gene relationships may constitute a first small step toward identifying (and possibly annotating) functional and process-related properties of subgraph patterns. Nonetheless, a number of individual THubs showed high preference for certain regulatory subgraph patterns, hinting at particular downstream cascade characteristics of THub control. For example, FFLs were preferred by *YLR013W (GAT3)* in the cell-cycle sub-network. There are four FFLs immediately downstream of this THub that can be aligned in a symmetrical grid (Figure 3A). Irrespective of whether these FFLs are coherent or incoherent, a highly complex mode of regulation can be generated through this module [18]. Theoretical analysis and experimental evidence suggest that autoregulation and cross-feedback have multiple functions in cell signaling systems [17]. FBLs consisting of three or more factors also enable similar functions in transcriptional regulatory networks [19,21]. In yeast transcriptional networks, FBLs with more than three regulators are relatively rare, but have been identified in high-throughput ChIP datasets [2,9].

There are only three instances of FBLs in the static network and the cell cycle sub-network, and even fewer in the other sub-networks; it may therefore be argued that the observed high-preference of the pattern could be caused by random fluctuation FBL. To address this issue, we introduced various kinds of noise to the networks, to tests the robustness on the THub subgraph preferences (see Protocol S1). Despite the low number of FBLs in the networks, the preference profiles were fairly stable against random perturbations, possibly owing (at least in part) to the fact that in both the static and cell cycle networks, the FBLs are interlinked in a larger structure (Figure 3B), which may have increased the structural stability of the networks. We therefore see little reason to assume that the THub preferences for FBLs are any less real than for other subgraph patterns.

A FBL-preferring THub, *YBR049C (REB1),* has been found to perform different biological functions under different conditions. Under endogenous conditions, it may work as a member of a clock or oscillator structure [22] to control multi-phase cell processes like cell cycle progression and sporulation. Under diauxic shift, however, it may work as a member of a switcher [21], or as a factor speeding up response times under DNA damage and stress response conditions [23,24]. Non-motif gene circuits have not yet been well-studied [19]. However, we found that under every condition there were a few THubs that preferred non-motif-like regulatory subgraph patterns, possibly indicating particular features of the signal transduction of these THubs. In the stress response network, the THub *YMR043W (Mcm1)* preferred two non-motif regulatory subgraph patterns, regulatory chain and multi-pooling regulation patterns. Responding to environmental stress, this THub might quickly pass the signal to the every corner of the network through these two patterns. When the stress is over, this may be sensed by persistent detector FFLs [1,18], and the signal can also be broadcasted through the same two non-motif-like regulatory patterns.

SIMs stand out as a peculiar case. Over-represented in most transcriptional network, SIMs have been regarded as important network building blocks conveying some sort of evolutionary advantage [1,3]. However, as SIMs were little, if at all, preferred by individual THubs, our results are unable to support the idea of particular position in the transcriptional network for this motif. As pointed out by Artzy-Randrup [25], network design biases, although not favoring any particular motif per se, may still produce an overabundance of particular subgraphs, and the reason for the global overrepresentation of SIMs may thus have to be sought elsewhere. Gene duplications are common evolutionary events and possibly the most important force driving network enlargement [26]. If gene duplication also includes the upstream control sequence, this will directly create a novel SIM. Such events are not rare [27]. Thus, the very mechanism of network expansion (e.g., gene duplication) may be sufficient to cause deviations from a true random network (i.e. overabundance of SIMs) without active selection for a particular motif per se (although it cannot be ruled out that gene duplication itself may be under positive selection). In this respect, the observed THub subgraph preferences may actually be seen as evidence in favor of an evolutionary explanation for the non-random distribution of subgraph motifs in transcriptional networks. Whereas it may be conceivable (although debatable [28]) that over-abundance of subgraphs in the biological networks may have other explanations than evolutionary selection [25], it seems utterly unlikely that there should at the same time exist some (non-evolutionary) network design “rule” able to account for individual transcription factors selectively accumulating one or the other subgraph motif, in particular, as this seems to occur independently of the overall network accumulation of motifs.

Distinguishing clearly between truly dynamic features and random fluctuations of a network remains a challenge. The SPL combines information on global topological organization with the connective structure of the network and relative abundances of local subgraphs, data which are all proven powerful tools for annotating gene expression data [29], inferring network mechanisms [30], and classifying networks into families [31]. The figurative representation of SPLs (e.g., Figure 1) is also a visualization of the inner structures of a regulatory network which could be used to obtain a clearer picture of network activity. This method for network subgraph analysis might also be applied to other biological or non-biological networks, such as metabolic networks, neuronal circuits, or electronic chips.

## Materials and Methods

### Dataset.

The S. cerevisiae transcriptional regulatory network data (see Table 5 for accession numbers of genes)*,* including the static and the five condition-specific (cell cycle, sporulation, diauxic shift, DNA damage, and stress response) networks, were retrieved from http://sandy.topnet.gersteinlab.org [9]. Auto-regulative interactions were excluded.

**Table 5:**
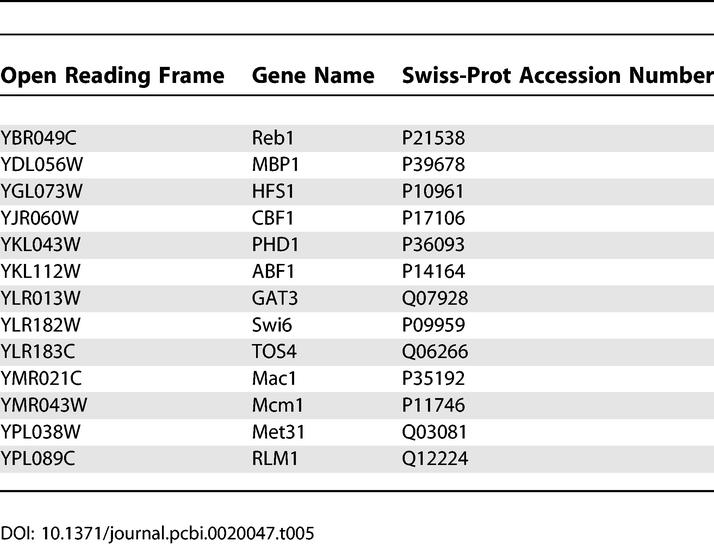
Swiss-Prot Accession Numbers for Genes and Proteins Mentioned in the Text

### THubs and the two classes of transcriptional regulatory patterns.

THubs were defined as transcription factors with more than 39 out-going degrees in the static transcription regulatory network, and with more than five out-going degrees in the condition specific sub-networks. To analyze the relationship between THubs and regulatory motifs, we selected two categories of basic regulatory subgraph patterns, the ring and the tree. Opening and closing are two of the basic topology characteristics of a subgraph pattern. Accordingly, a subgraph pattern is said to be a ring if, and only if, it represents a single loop, regardless of the directions of the edges. A subgraph pattern is said to be a tree if, and only if, it contains no ring as a component (Figure 1). Only trees and rings with three or four vertices were considered because, at the 2-vertex level, T2–1 is trivial, whereas R2–1 is too infrequent to be considered independently. On the other hand, due to computational limitations, it is difficult to identify all the rings and trees in the network when the number of vertices in the subgraphs exceeds four. An additional advantage of these subgraph sets is that there are no overlaps within a set of subgraphs with a fixed number of vertices, and also that these subgraph sets are basic enough for our analysis.

### Definition of THub SPP and SPL.

In this work, we use term SPP to describe the relative abundances of a set of regulatory subgraph patterns that appear in the cascade downstream of a transcription factor. Given a subgraph pattern, *P,* and a transcription factor, *H,* we count the weighed abundance of subgraphs connected to the transcription factor as:

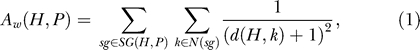
where *SG(H_,_ P)* is a set that includes all the subgraph patterns *P* appearing in the cascade downstream of the transcription factor H. sg is a member of set *SG(H, P)*. *N(sg)* is the set of nodes in *sg*. *d(H, k)* is the length of the shortest path from transcription factor *H* to the node *k* in the network, calculated by *Dijkstra*'s algorithm [32]. The weigh factor in Equation 1, *1/(d(H, k) + 1)^2^*, was designed to quantify the reduction in “signal strength” with increasing distance from a transcription factor. However, in addition to the number in nodes of the transcription factors downstream cascade, there are several other systematic biases influencing the abundance calculation. The transcriptional patterns appear in the network with a wide range of frequencies [1,3], and the topological relationships between different subgraph patterns may also introduce systematic bias. To account for all the above considerations, we normalized the abundance for all chosen subgraph patterns of the THubs by


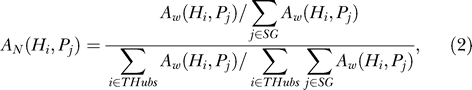


where *THubs* denotes all THubs in a transcriptional regulatory network and *SG* denotes a set of subgraph patterns. After the normalization process, we obtained the “normalized abundances” of all the given THubs and subgraphs. The normalized abundance of a subgraph pattern was called the THub SPP for this pattern. The SPP of a transcription factor was then defined as the vector of the normalized abundances of all subgraphs in *SG* and, in turn, the SPL of a network was defined as the collection of SPPs of all the THubs in the transcriptional regulatory network. The SPL can be represented as a matrix where all subgraph normalized abundances are laid out in certain order for every THubs (Figure 2A).

### Statistical significance and robustness analysis.

Randomly shuffled networks were generated to assess the statistical significance of our analysis. We also tested the robustness of our results from different source of noises and variations. The detailed algorithm for noise generation and assessment for the robustness can be found in Protocol S1.

## Supporting Information

Protocol S1 Supporting Methods, Figures, and Tables (8.0 MB TXT)

## Abbreviations

FBL: feedback loop
FFL: feed-forward loop
SIM: single input motif
SPL: subgraph preference landscape
SPP: subgraph preference profile
THub: transcription hub

## References

[pcbi-0020047-b001] Shen-Orr SS, Milo R, Mangan S, Alon U (2002). Network motifs in the transcriptional regulation network of Escherichia coli. Nat Genet.

[pcbi-0020047-b002] Lee TI, Rinaldi NJ, Robert F, Odom DT, Bar-Joseph Z (2002). Transcriptional regulatory networks in Saccharomyces cerevisiae. Science.

[pcbi-0020047-b003] Milo R, Shen-Orr S, Itzkovitz S, Kashtan N, Chclovskii D (2002). Network motifs: Simple building blocks of complex networks. Science.

[pcbi-0020047-b004] Vazquez A, Dobrin R, Sergi D, Eckmann JP, Oltvai ZN (2004). The topological relationship between the large-scale attributes and local interaction patterns of complex networks. Proc Natl Acad Sci U S A.

[pcbi-0020047-b005] Guelzim N, Bottani S, Bourgnie P, Kepes F (2002). Topological and causal structure of the yeast transcriptional regulatory network. Nat Genet.

[pcbi-0020047-b006] Yu H, Greenbaum D, Xin Lu H, Zhu X, Gerstein M (2004). Genomic analysis of essentiality within protein networks. Trends Genet.

[pcbi-0020047-b007] Bray D (1995). Protein molecules as computational elements in living cells. Nature.

[pcbi-0020047-b008] Ma'ayan A, Jenkins SL, Neves S, Hasseldine A, Grace E (2005). Formation of regulatory patterns during signal propagation in a Mammalian cellular network. Science.

[pcbi-0020047-b009] Luscombe NM, Babu MM, Yu H, Snyder M, Teichmann SA (2004). Genomic analysis of regulatory network dynamics reveals large topological changes. Nature.

[pcbi-0020047-b010] Barabasi AL, Oltvai ZN (2004). Network biology: Understanding the cell's functional organization. Nat Rev Genet.

[pcbi-0020047-b011] Ma HW, Buer J, Zeng AP (2004). Hierarchical structure and modules in the Escherichia coli transcriptional regulatory network revealed by a new top-down approach. BMC Bioinformatics.

[pcbi-0020047-b012] Ma HW, Kumar B, Ditges U, Gunzer F, Buer J (2004). An extended transcriptional regulatory network of Escherichia coli and analysis of its hierarchical structure and network motifs. Nucleic Acids Res.

[pcbi-0020047-b013] Morrow BE, Johnson SP, Warner JR (1989). Proteins that bind to the yeast rDNA enhancer. J Biol Chem.

[pcbi-0020047-b014] Elble R, Tye BK (1991). Both activation and repression of a-mating-type-specific genes in yeast require transcription factor Mcm1. Proc Natl Acad Sci U S A.

[pcbi-0020047-b015] Ashburner M, Ball CA, Blake JA, Botstein D, Butler H (2000). Gene ontology: Tool for the unification of biology. The Gene Ontology Consortium. Nat Genet.

[pcbi-0020047-b016] Thomas D, Jacquemin I, Surdin-Kerjan Y (1992). MET4, a leucine zipper protein, and centromere-binding factor 1 are both required for transcriptional activation of sulfur metabolism in Saccharomyces cerevisiae. Mol Cell Biol.

[pcbi-0020047-b017] Wolf DM, Arkin AP (2003). Motifs, modules, and games in bacteria. Curr Opin Microbiol.

[pcbi-0020047-b018] Mangan S, Alon U (2003). Structure and function of the feed-forward loop network motif. Proc Natl Acad Sci U S A.

[pcbi-0020047-b019] Wall ME, Hlavacek WS, Savageau MA (2004). Design of gene circuits: Lessons from bacteria. Nat Rev Genet.

[pcbi-0020047-b020] Babu MM, Luscombe NM, Aravind L, Gerstein M, Teichmann SA (2004). Structure and evolution of transcriptional regulatory networks. Curr Opin Struct Biol.

[pcbi-0020047-b021] Ferrell JE (2002). Self-perpetuating states in signal transduction: Positive feedback, double-negative feedback, and bistability. Curr Opin Cell Bio.

[pcbi-0020047-b022] Bar-Or RL, Maya R, Segel LA, Alon U, Levine AJ (2000). Generation of oscillations by the p53-Mdm2 feedback loop: A theoretical and experimental study. Proc Natl Acad Sci U S A.

[pcbi-0020047-b023] Egan SM, Schleif RF (1993). A regulatory cascade in the induction of rhaBAD. J Mol Biol.

[pcbi-0020047-b024] Via P, Badia J, Baldoma L, Obradors N, Aguilar J (1996). Transcriptional regulation of the Escherichia coli rhaT gene. Microbiology.

[pcbi-0020047-b025] Artzy-Randrup Y, Fleishman SJ, Ben-Tal N, Stone L (2004). Comment on “Network motifs: simple building blocks of complex networks” and “Superfamilies of evolved and designed networks.”. Science.

[pcbi-0020047-b026] Brenner SE, Hubbard T, Murzin A, Chothia C (1995). Gene duplications in H. influenzae. Nature.

[pcbi-0020047-b027] Teichmann SA, Babu MM (2004). Gene regulatory network growth by duplication. Nat Genet.

[pcbi-0020047-b028] Milo R, Shen-Orr S, Itzkovitz S, Kashtan N, Chclovskii D (2004). Response to comment on “Network motifs: Simple building blocks of complex networks” and “Superfamilies of evolved and designed networks.”. Science.

[pcbi-0020047-b029] Zhou X, Kao MC, Wong WH (2002). Transitive functional annotation by shortest-path analysis of gene expression data. Proc Natl Acad Sci U S A.

[pcbi-0020047-b030] Middendorf M, Ziv E, Wiggins CH (2005). Inferring network mechanisms: The Drosophila melanogaster protein interaction network. Proc Natl Acad Sci U S A.

[pcbi-0020047-b031] Kashtan N, Itzkovitz S, Milo R, Alon U (2004). Efficient sampling algorithm for estimating subgraph concentrations and detecting network motifs. Bioinformatics.

[pcbi-0020047-b032] Dijkstra EW (1959). A note on two problems in connexion with graphs. Numerische Mathematik.

